# Molecular determinants of SR-B1-dependent *Plasmodium* sporozoite entry into hepatocytes

**DOI:** 10.1038/s41598-020-70468-2

**Published:** 2020-08-11

**Authors:** Anne-Claire Langlois, Giulia Manzoni, Laetitia Vincensini, Romain Coppée, Carine Marinach, Maryse Guérin, Thierry Huby, Véronique Carrière, François-Loïc Cosset, Marlène Dreux, Eric Rubinstein, Olivier Silvie

**Affiliations:** 1 Sorbonne Université, INSERM, CNRS, Centre d’Immunologie et des Maladies Infectieuses, CIMI-Paris, 75013 Paris, France; 2 Université de Paris, UMR 261 MERIT, IRD, 75006 Paris, France; 3 Sorbonne Université, INSERM, Unité de Recherche sur les Maladies Cardiovasculaires et Métaboliques, 75013 Paris, France; 4 Sorbonne Université, INSERM, Centre de Recherche de St-Antoine, 75012 Paris, France; 5 CIRI – Centre International de Recherche en Infectiologie, Univ Lyon, Université Claude Bernard Lyon 1, Inserm, U1111, CNRS, UMR5308, ENS Lyon, 69007 Lyon, France

**Keywords:** Parasite biology, Malaria

## Abstract

Sporozoite forms of the *Plasmodium* parasite, the causative agent of malaria, are transmitted by mosquitoes and first infect the liver for an initial round of replication before parasite proliferation in the blood. The molecular mechanisms involved during sporozoite invasion of hepatocytes remain poorly understood. Two receptors of the Hepatitis C virus (HCV), the tetraspanin CD81 and the scavenger receptor class B type 1 (SR-B1), play an important role during the entry of *Plasmodium* sporozoites into hepatocytes. In contrast to HCV entry, which requires both CD81 and SR-B1 together with additional host factors, CD81 and SR-B1 operate independently during malaria liver infection. Sporozoites from human-infecting *P. falciparum* and *P. vivax* rely respectively on CD81 or SR-B1. Rodent-infecting *P. berghei* can use SR-B1 to infect host cells as an alternative pathway to CD81, providing a tractable model to investigate the role of SR-B1 during *Plasmodium* liver infection. Here we show that mouse SR-B1 is less functional as compared to human SR-B1 during *P. berghei* infection. We took advantage of this functional difference to investigate the structural determinants of SR-B1 required for infection. Using a structure-guided strategy and chimeric mouse/human SR-B1 constructs, we could map the functional region of human SR-B1 within apical loops, suggesting that this region of the protein may play a crucial role for interaction of sporozoite ligands with host cells and thus the very first step of *Plasmodium* infection.

## Introduction

Despite progress in malaria control over the last two decades, *Plasmodium* parasites continue to cause more than 200 million cases every year^1^. After their inoculation into the skin by infected *Anopheles* mosquitoes, *Plasmodium* sporozoites rapidly migrate through tissues and blood vessels to reach the liver, using active gliding motility and cell traversal activity. Once in the liver, they first traverse hepatocytes before invading them and developing into exo-erythrocytic forms (EEFs), surrounded by a parasitophorous vacuole (PV) membrane. Inside the PV, they differentiate into thousands of merozoites, which are eventually packed in merosomes and released into the blood circulation, where they invade red blood cells, provoking the symptomatic phase of the disease.

Several host and parasite factors implicated in sporozoite invasion have been identified but the underlying molecular interactions remain unknown. Human and murine parasites share similar invasion routes, with two distinct invasion pathways that depend on the tetraspanin CD81 or the scavenger receptor class B type 1 (SR-B1)^2–5^. The human parasite *P. falciparum* and the murine parasite *P. yoelii* both require CD81^3^, whereas *P. vivax* enters human hepatocytes using SR-B1^4^*.* Interestingly, the murine parasite *P. berghei* can invade cells in vitro using either CD81 or, alternatively, a SR-B1-dependent route in the absence of CD81^4^. Whilst SR-B1 is the only known hepatocyte entry factor for *P. vivax* sporozoites, studying this parasite remains difficult, notably due to the limited access to infected mosquitoes. In this context, *P. berghei* provides an attractive model to investigate the role of SR-B1 during sporozoite infection.

SR-B1 is a highly glycosylated transmembrane protein that belongs to the CD36 family, which also includes CD36 and the lysosomal integral membrane protein 2 (LIMP-2). A tertiary structure of SR-B1 was predicted using LIMP-2 crystal structure as a template^6^. SR-B1 possesses two transmembrane regions, cytoplasmic N- and C-termini, and a large extracellular domain constituted by a ß-strand tunnel topped by a helical bundle^6, 7^. SR-B1 apical helices are involved in the binding of high density lipoproteins (HDLs)^8^. The hydrophobic cavity traversing the entire protein is implicated in a selective lipid transfer with cholesteryl ester bidirectional exchanges between HDLs and the cell membrane^8, 9^.

In this study, we show that murine SR-B1 poorly supports *P. berghei* infection as compared to its human counterpart. We took advantage of this functional difference to study the structural determinants of the SR-B1 receptor in *Plasmodium* invasion, using a structure-guided strategy based on chimeric constructs combining mouse and human SR-B1 domains*.*

## Results

### Blockade of CD81 does not prevent *P. berghei* infection in SR-B1-deficient primary mouse hepatocytes

**Figure 1:**
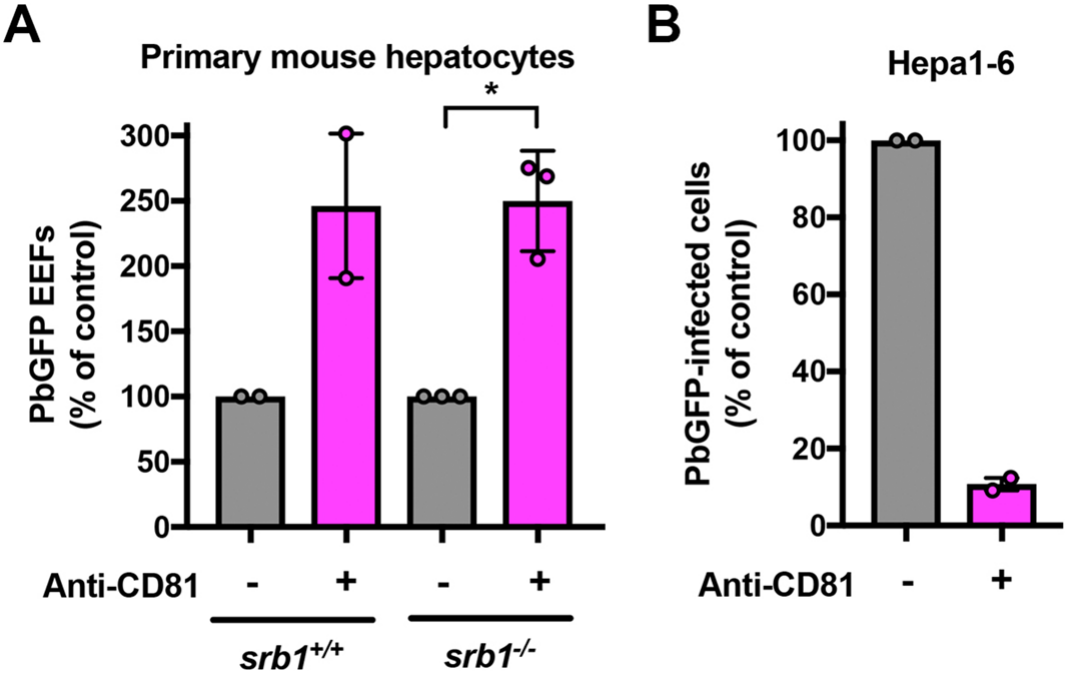
*P. berghei* sporozoites can infect primary mouse hepatocytes through CD81-and SR-B1-independent pathways. (**A**) Primary hepatocytes isolated from WT or SR-B1 deficient C57BL/6 mice were infected with PbGFP sporozoites in the absence or presence of neutralizing anti-mCD81 mAb MT81, and cultured for 24 h before EEF quantification (mean control values for each experiment without MT81: 63 and 266 EEFs/well in *srb1*^+/+^ hepatocytes; 94, 311 and 420 EEFs/well in *srb1*^−/−^ hepatocytes). *p < 0.05 (ratio paired *t* test). **(B)***P. berghei* infection in Hepa1-6 cells was inhibited by the anti-mCD81 mAb MT81 antibody (mean control values for each experiment without MT81: 0.64 and 1.09% PbGFP-infected cells).

*P. berghei* sporozoites infect human hepatocyte cell lines using CD81 or SR-B1 as alternative entry routes^4^. Previous studies have shown that mice deficient for either CD81 or SR-B1 remain susceptible to *P. berghei* sporozoite infection^2, 3, 5^, which could be explained by the mutual functional compensation between the two entry routes^4^. To test whether CD81 and SR-B1 are the only host factors permitting the entry of the parasite in murine hepatocytes, we analyzed the effect of CD81 neutralization in primary hepatocytes isolated from wild type (WT) or transgenic C57BL/6 J mice harboring a Cre-mediated SR-B1 gene inactivation specifically in the liver^10^. We used the anti-CD81 monoclonal antibody MT81 to neutralize the CD81-dependent entry pathway^11^. CD81 inhibition did not impede *P. berghei* infection of SR-B1-deficient hepatocytes, but, paradoxically, substantially increased the infection rate, similarly to WT hepatocytes (Fig. 1a). This enhancing effect of anti-CD81 antibodies on *P. berghei*-infection has been reported before in C57BL/6 mouse hepatocyte cultures, yet the underlying mechanism remains unknown^12^. MT81 efficiently blocked *P. berghei* sporozoite infection in Hepa1-6 hepatoma cells (Fig. 1b), confirming the neutralizing activity of the antibody^12^. These data indicate that *P. berghei* sporozoites can infect primary mouse hepatocytes in a CD81- and SR-B1-independent manner and suggest the role of additional entry factors. However, it is difficult to conclude from these experiments on the contribution of mouse SR-B1 in *P. berghei* sporozoite entry.

### CRISPR-Cas 9 mediated inactivation of CD81 abrogates *P. berghei* infection in Hepa1-6 cells

**Figure 2:**
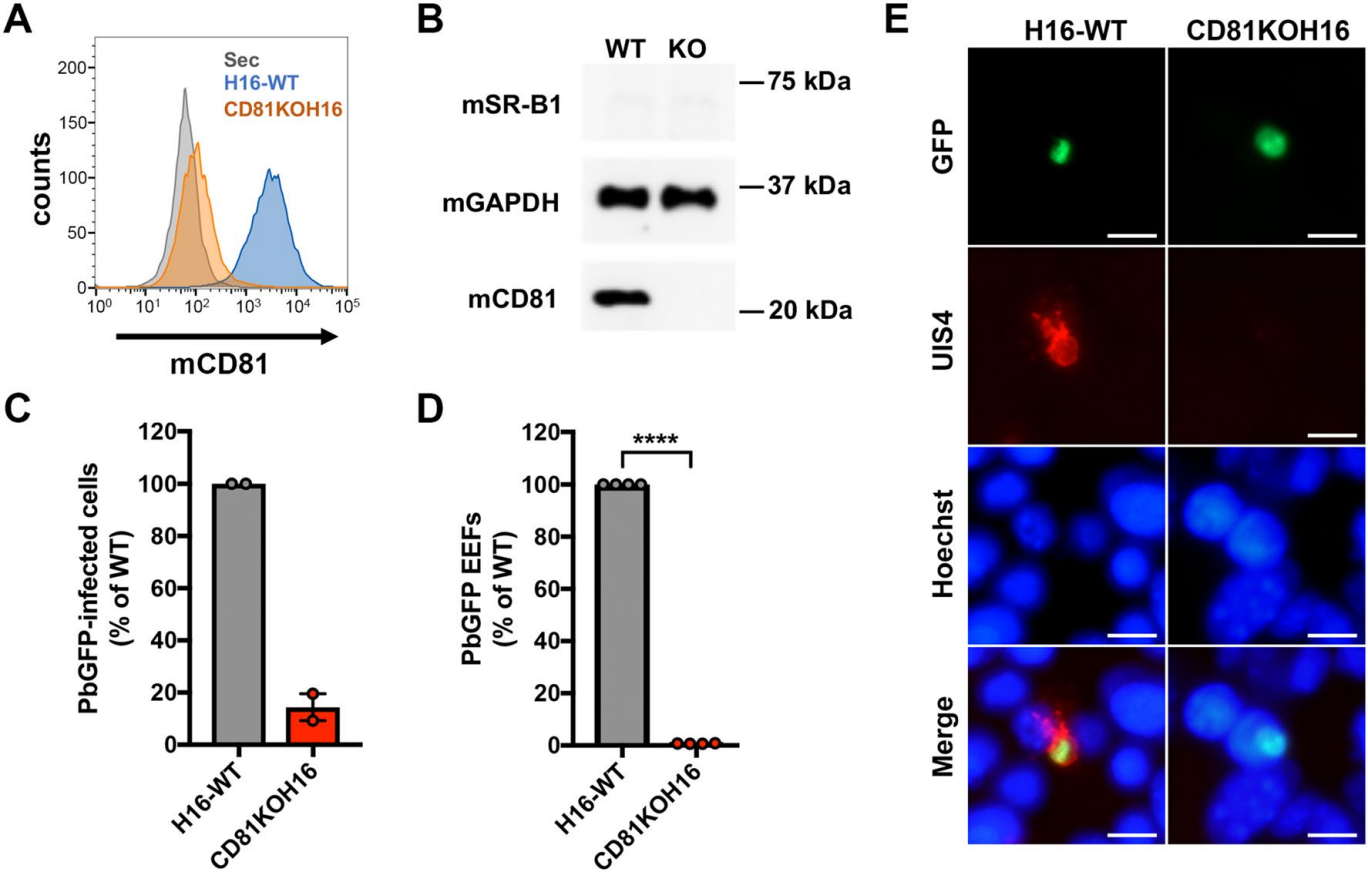
CRISPR-mediated inactivation of CD81 abrogates *P. berghei* infection in Hepa1-6 cells. **(A)** Hepa1-6 and CD81KOH16 cells were stained for surface CD81 with anti-CD81 MT81 monoclonal antibody and Alexa Fluor 488-conjugated secondary antibodies, before flow cytometry analysis. Histograms represent the fluorescence intensity of extracellular CD81 proteins for WT Hepa1-6 (blue) and CD81KOH16 cells (orange). The grey histogram represents cells stained with secondary antibodies only (Control). **(B)** Western blot analysis of total CD81 protein expression in WT Hepa1-6 and CD81KOH16 cells. GAPDH was used as loading control. **(C–E)** WT Hepa1-6 and CD81KOH16 cells were infected with PbGFP sporozoites and analyzed 24 h after invasion by flow cytometry **(C)** or microscopy **(D, E)** after staining with anti-UIS4 antibodies (red) and Hoechst 33342 nuclear stain (blue). The mean control values for each experiment were 0.27 and 0.93% PbGFP-infected cells (**C**), and 144, 145, 215 and 288 EEFs/well (**D**). ****p < 0.0001 (ratio paired *t* test). The images show PbGFP EEFs (green) surrounded by a UIS4-positive PV membrane (red) or intranuclear parasites in CD81KOH16 cells. Scale bar, 10 μm.

To further analyze the role of human and mouse SR-B1, we took advantage of the fact that the murine hepatoma Hepa1-6 cells do not express SR-B1^4^ and generated a variant cell line deficient for murine CD81 (CD81 knockout (KO) Hepa1-6 or CD81KOH16) using the CRISPR-Cas9 system. The loss of CD81 expression in CD81KOH16 cells was confirmed by flow cytometry (Fig. 2a) and western blot (Fig. 2b). Confirming previous results using either antibodies or small interfering RNA (siRNA), a dramatic reduction of the percentage of *P. berghei* infected cells was observed in the CD81KOH16 cell line as compared to parental cells (Fig. 2c). PV quantification by microscopy after staining of UIS4, a PV membrane marker^13^, revealed a complete inhibition of productive infection in CD81KOH16 cells (Fig. 2d). Intranuclear UIS4-negative parasites were observed in the CD81-deficient cells, contrasting with the well-developed EEFs with a strong UIS4 staining found in the parental Hepa1-6 cells (Fig. 2e). We have shown before that intranuclear parasites result from sporozoites arrested during cell traversal^14^. The residual intracellular parasite population observed by flow cytometry in the KO cells (Fig. 2c) thus likely corresponds to non-productive invasion associated with cell traversal. The CD81KOH16 cell line, which lacks CD81 and has lost susceptibility to *P. berghei* infection, thus provides a suitable tool to investigate SR-B1 function through genetic complementation experiments.

### Human and murine SR-B1 differ in their ability to support *P. berghei* sporozoite infection

**Figure 3:**
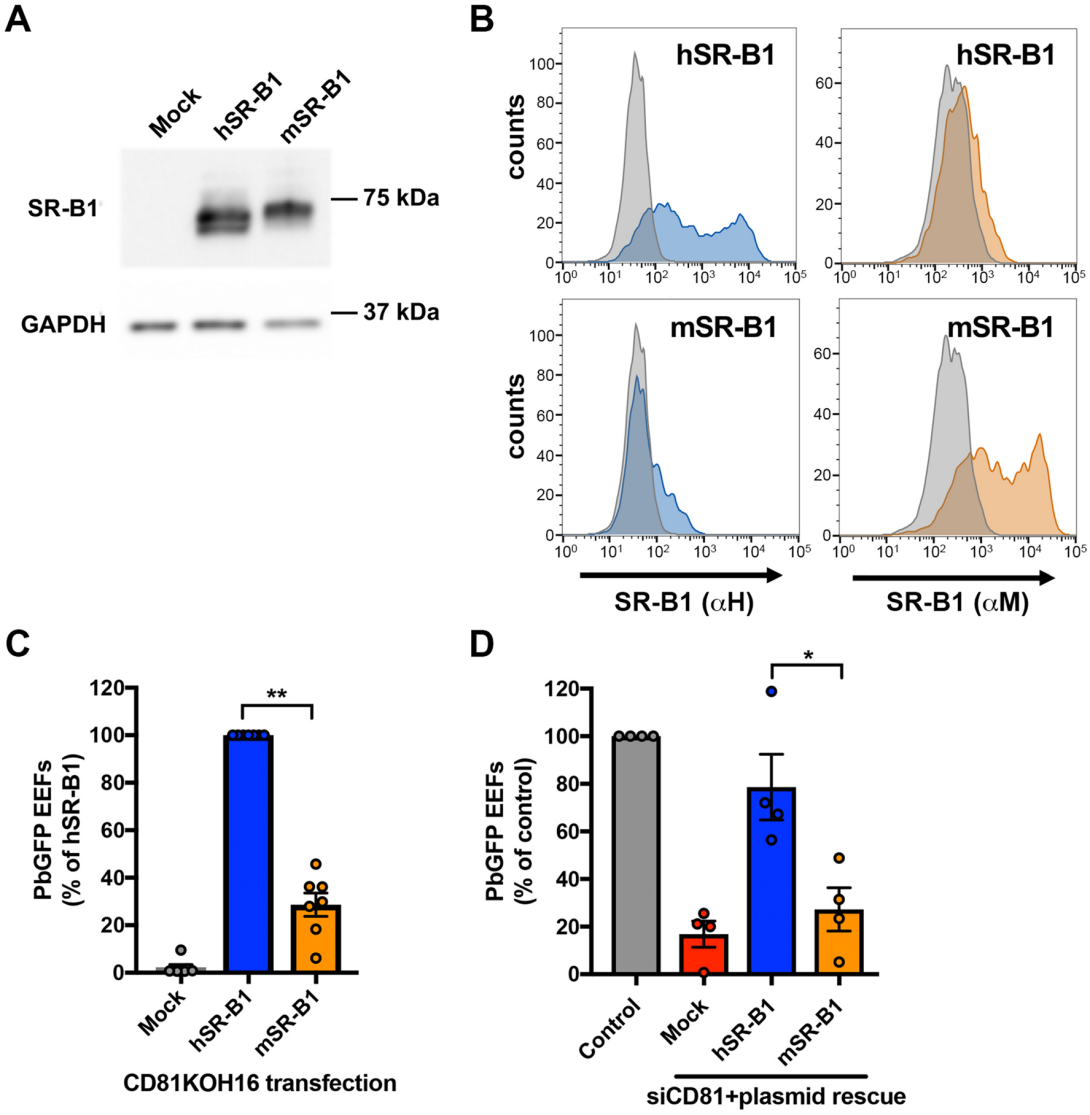
Mouse SR-B1 poorly supports *P. berghei* sporozoite invasion. **(A,B)** CD81KOH16 cells were transfected with either mouse or human SR-B1 plasmids, or no plasmid as a control (Mock). Total protein expression was analyzed using polyclonal anti-SR-B1 antibodies (Ab24603) by western blot **(A)** with GAPDH as a loading control. Surface protein expression was analyzed by flow cytometry **(B)** using anti-human “αH” SR-B1 polyclonal rabbit serum (blue) and anti-mouse “αM” polyclonal antibodies NB400-113 (orange). The grey histogram represents untransfected cells labeled with the corresponding antibody. **(C,D)** CD81KOH16 **(C)** and WT Hepa1-6 cells treated with siRNA against CD81 24 h before **(D)**, were transfected with mouse or human SR-B1 plasmids, or no plasmid as a negative control (Mock), and then infected with PbGFP sporozoites. EEF numbers were counted by microscopy after UIS4 staining, 24 h after sporozoite addition. The mean control values for each experiment were 59, 139, 214, 245, 299, 315 and 383 EEFs/well in hSR-B1-transfected CD81KOH16 cells **(C)**, and 30, 140, 155 and 215 EEFs/well in control Hepa1-6 cells **(D)**. *p < 0.05; **p < 0.01 (repeated measures one-way ANOVA followed by Tukey’s multiple comparisons test).

We have previously shown that the ectopic expression of human SR-B1 can restore *P. berghei* infection in Hepa1-6 cells where CD81 expression has been previously silenced with siRNA^4^. Here, we compared the functionality of SR-B1 proteins from human and mouse origins (hereinafter referred to as hSR-B1 and mSR-B1, respectively) during *P. berghei* infection after genetic complementation of CD81KOH16 cells. After transient cell transfection with plasmids encoding hSR-B1 or mSR-B1, we observed a similar expression of the two proteins by western blot (Fig. 3a) and flow cytometry (Fig. 3b). The transfected cells were then infected with GFP-expressing *P. berghei* sporozoites (PbGFP). In agreement with our previous observations in CD81-silenced cells^4^, the transfection of hSR-B1 in CD81KOH16 cells restored their susceptibility to *P. berghei* infection (Fig. 3c). Unexpectedly, despite similar protein expression, mSR-B1 was not as efficient as hSR-B1 in restoring *P. berghei* infection (Fig. 3c). We performed similar transfection experiments in the parental Hepa1-6 cell line after CD81 silencing with siRNA, which confirmed the lower functionality of mSR-B1 protein during *P. berghei* sporozoite infection as compared to hSR-B1 (Fig. 3d).

### Human and mouse SR-B1 protein sequence analysis and structure-homology modeling

**Figure 4:**
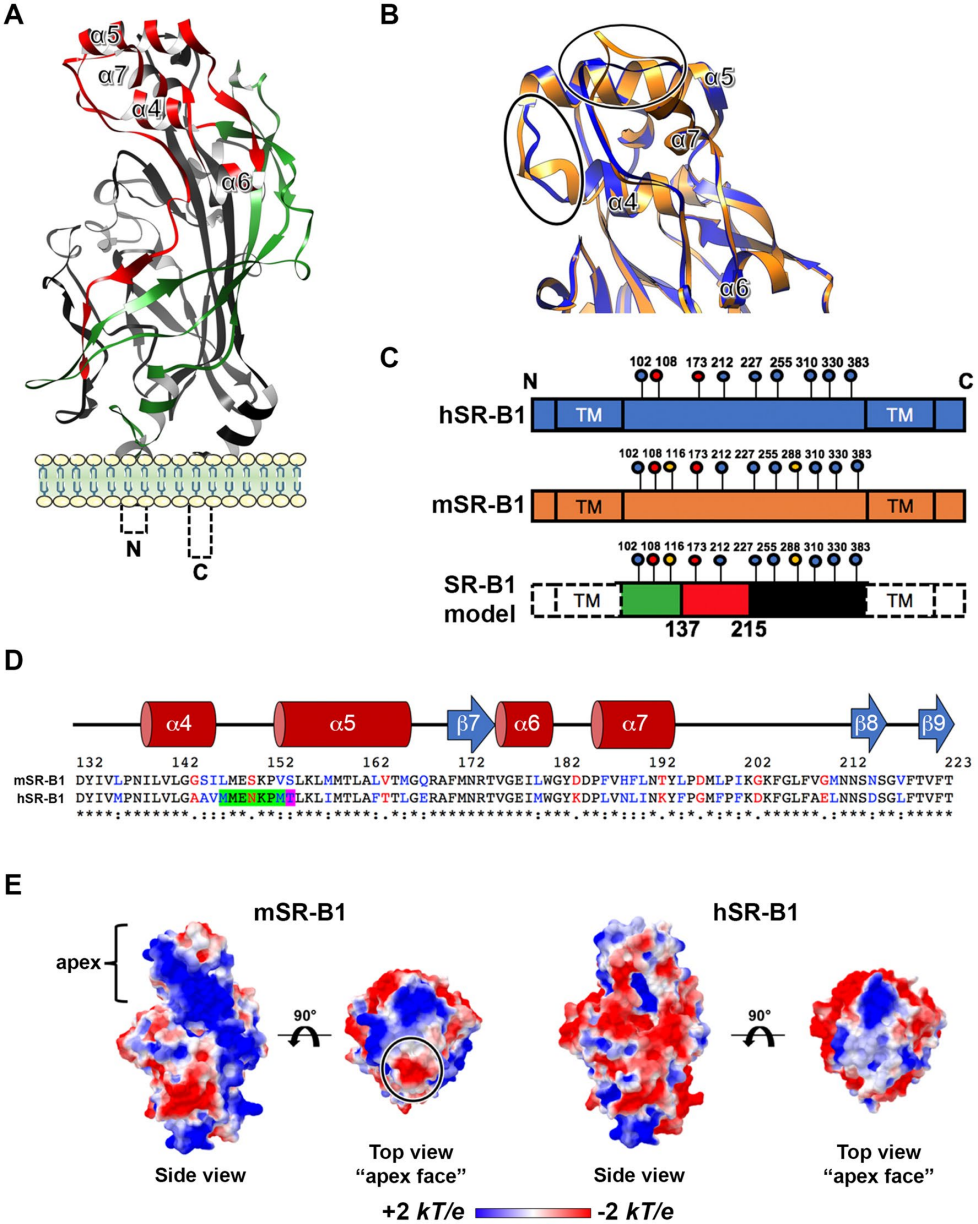
SR-B1 modeling identifies potential functional regions. **(A)** Predicted tertiary structure of hSR-B1 extracellular domain by homology modeling using CD36 (PDB ID: 5lgd) as a template, with the three regions referred to as “N-terminal” (green), “apex” (red) and “C-terminal” (black). **(B)** A close-up view of structural alignment of the apical helix bundle of mouse (orange) and human (blue) SR-B1, with their four alpha helices (α4 to α7). The main structural differences are circled in black. **(C)** Schematic representation of SR-B1 N-glycosylation sites on human (blue) and mouse (orange) proteins. Two determinant sites for SR-B1 structure and function are in red (Asn 108 and 173), mouse specific sites are in yellow (Asn 116 and 288) and conserved sites are in blue. SR-B1 model is a schematic representation of the delineated regions (“N-terminal” (green), “apex” (red), “C-terminal” (black)) in SR-B1 protein displaying all potential N-glycosylation sites. **(D)** Pairwise sequence alignment of mSR-B1 and hSR-B1 proteins for the 132–223 apical region with corresponding predicted human secondary structure (alpha helices in red and beta strand in blue). Identical, similar and different amino acids are represented in black, blue and red respectively. The threonine residue position corresponding to PfEMP1 binding phenylalanine in CD36 homolog is highlighted in purple. The residues in SR-B1 equivalent to Enterovirus-interacting site in LIMP-2 are highlighted in green and purple. **(E)** Electrostatic surface potential of mSR-B1 and hSR-B1 extracellular domain from side and top views. Values are in units of kT/e at 298 K, on a scale of − 2 kT/e (red) to + 2 kT/e (blue). White color indicates a neutral potential. The black circle highlights a differential electrostatic surface potential between mSR-B1 and hSR-B1 at the top of the “apex” region.

We next investigated the structural basis that could explain the differential functionality of human and mouse SR-B1 during *P. berghei* sporozoite invasion. hSR-B1 (isoform 1) contains 509 amino acids (AA) and presents a large extracellular domain (404 AA) flanked by two transmembrane domains (both 23 AA) and two cytoplasmic tails (N-terminal: 12 AA; C-terminal: 47 AA)^6^. The modeling of hSR-B1 using CD36 as a template (PDB ID: 5lgd)^7^ shows that the extracellular part of the receptor can be divided into three regions: a N-terminal region (AA 36–136) harboring a thrombospondin-binding domain in the homologous CD36 protein^15^, an apical region (AA 137–214) consisting of four alpha helices (α4, 5, 6 and 7), and a large C-terminal region (AA 215–439) contributing to the hydrophobic channel (Fig. 4a,c). The pairwise sequence alignment of hSR-B1 and mSR-B1 showed that the N-terminal and C-terminal extracellular regions were the most similar, with 81.1% and 85.7% identity, respectively, whilst the apical domain is more divergent, with 66.2% identity (Fig. 4c,d). The hSR-B1 protein harbors 9 N-glycosylation sites, against 11 sites for mSR-B1 (Fig. 4c), which likely explains the different migration pattern observed in western blots^16^ (Fig. 3a). The superposition of hSR-B1 and mSR-B1 structural models suggested differences for two loops at the very top of the apex, between the α4 and α5 helices and after the α7 helix (Fig. 4b). The model also suggested differences in the electrostatic surface potentials in this area (Fig. 4e). When the structure is orientated in a side view to present its hydrophobic tunnel entrance, the apex lateral surface of mSR-B1 seems to be mainly electropositive whereas electronegativity is predominant in the human model (Fig. 4e). Remarkably, whilst in the model the top of the apical surface is strictly neutral to electropositive in hSR-B1, mSR-B1 displays a dense electronegative region (Fig. 4e), notably due to the presence of an aspartate residue at position 197 (Fig. 4d).

### The apical domain of SR-B1 plays a crucial role during *P. berghei* infection

**Figure 5:**
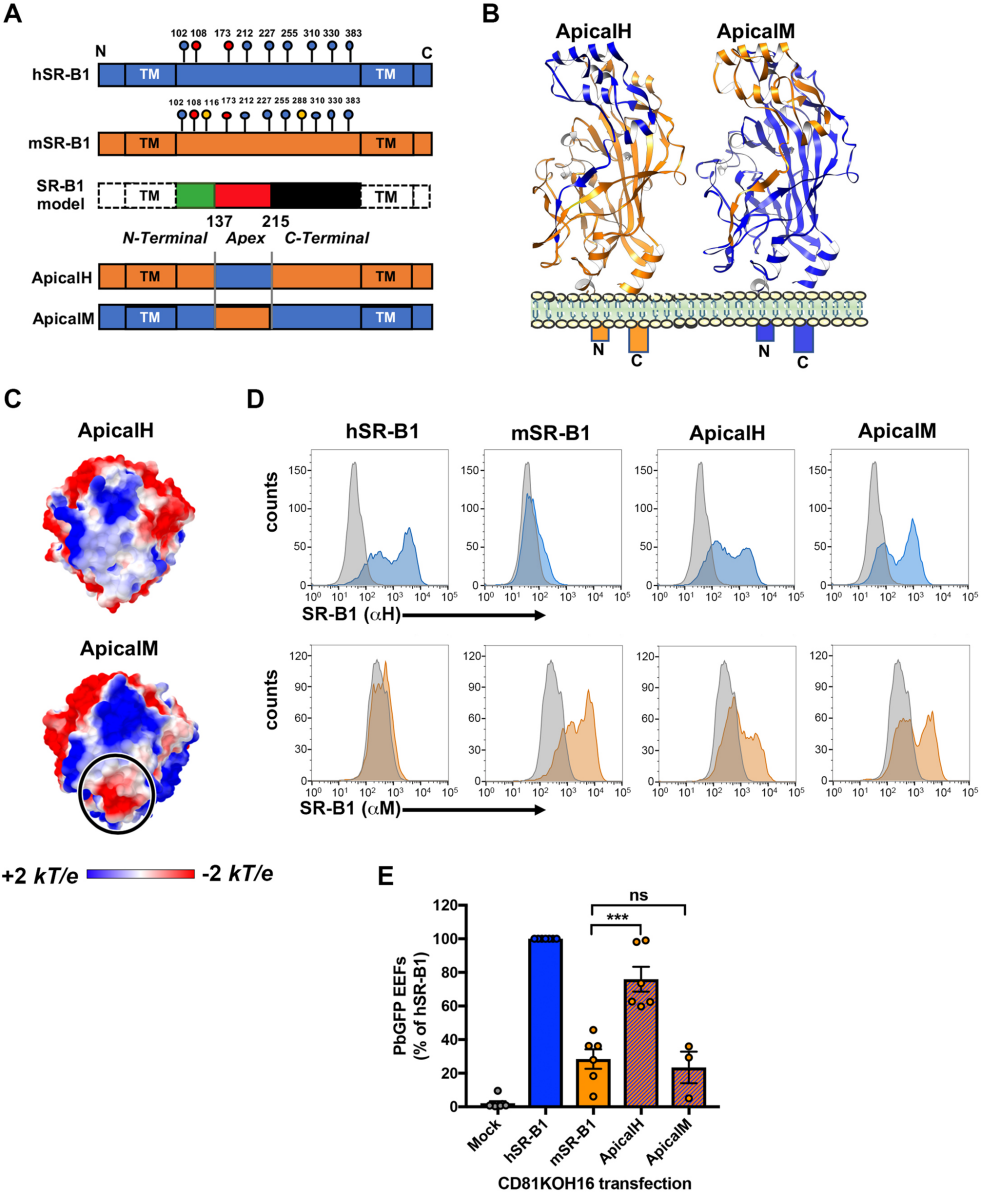
The apical domain of SR-B1 plays a crucial role during *P. berghei* infection. **(A)** Schematic representation of the ApicalH and ApicalM chimeric constructs. **(B)** Predicted tertiary structure of ApicalH and ApicalM chimeras by homology modeling, highlighting the portions of mouse (orange) or human (blue) origins. **(C)** Top views of the electrostatic surface potential of ApicalH and ApicalM chimeras’ apex. Values are in units of kT/e at 298 K, on a scale of − 2 kT/e (red) to + 2 kT/e (blue). White color indicates a neutral potential. The black circle highlights a differential electrostatic surface potential between the two chimeric constructs at the top of the “apex” region. **(D)** CD81KOH16 cells were transfected with hSR-B1, mSR-B1, ApicalH or ApicalM chimera plasmids, or no plasmid as a control (Mock). Protein surface expression was analyzed using anti-hSR-B1 (“αH”, blue histograms) and anti-mSR-B1 (“αM”, orange histograms), 24 h after transfection. The grey histogram represents untransfected cells stained with the cognate antibody. **(E)** CD81KOH16 cells were transfected with hSR-B1, mSR-B1, ApicalH or ApicalM constructs, or no plasmid as a control (Mock), and infected with PbGFP sporozoites 24 h after transfection. The number of infected cells (EEFs) was determined by microscopy after UIS4 staining, 24 h after sporozoite addition (mean control values for each experiment: 59, 139, 214, 245, 299, 315 and 383 EEFs/well in hSR-B1-transfected CD81KOH16 cells). ns, non-significant; ***p < 0.001 (one-way ANOVA followed by Tukey’s multiple comparisons test).

To determine whether the predicted structural differences at the apical domain of SR-B1 could explain the differential functionality of human and mouse SR-B1, we analyzed the functional properties of two chimeric constructs made of human and mouse sequences of SR-B1. The ApicalH chimera corresponds to a mSR-B1 backbone protein with a human apical region (AA 137–214) (Fig. 5a,b). Reciprocally, the ApicalM chimera corresponds to a hSR-B1 protein bearing a murine Apical region (Fig. 5a,b). The electrostatic surface potentials of ApicalH and ApicalM apex top are similar to human and mouse SR-B1, respectively, with only ApicalM showing a dense electronegative spot (Fig. 5c). CD81KOH16 cells were transiently transfected with plasmids encoding hSR-B1, mSR-B1, ApicalH or ApicalM. The two chimeras were expressed at the surface of transfected cells and detected by flow cytometry using anti-human and anti-mouse SR-B1 polyclonal antibodies (Fig. 5d). They were also detected by western blot analysis of whole cellular extracts (Fig S1). A slightly higher band was observed in the lanes corresponding to cells transfected with mSR-B1 and ApicalH constructs compared to hSR-B1 and ApicalM, likely explained by the differential glycosylation pattern of the mSR-B1 backbone^16^ (Fig. 5a). Cells transfected with ApicalH and ApicalM constructs bound Cy5-labelled HDLs (Fig S2), similarly to hSR-B1 and mSR-B1, suggesting that both chimeras are correctly folded.

Transfected cells were then incubated with *P. berghei* sporozoites, and the number of infected cells was determined at 24 h post-infection. These experiments revealed that replacement of the apex of mSR-B1 by that of hSR-B1 in ApicalH yielded a chimera with a 2–3 fold increase in *P. berghei* infection rates as compared to mSR-B1 (Fig. 5e). Reciprocally, replacement of the apex of hSR-B1 by that of mSR-B1 in the ApicalM chimera resulted in a loss of function, with infection levels similar to those observed after transfection of mSR-B1 (Fig. 5e). Altogether, these results demonstrate that the hSR-B1 apical helix bundle (AA 137–214) is functionally determinant during *P. berghei* sporozoite invasion of hepatocytic cell lines.

### A short portion of the apical domain of hSR-B1 facilitates *P. berghei* infection

**Figure 6:**
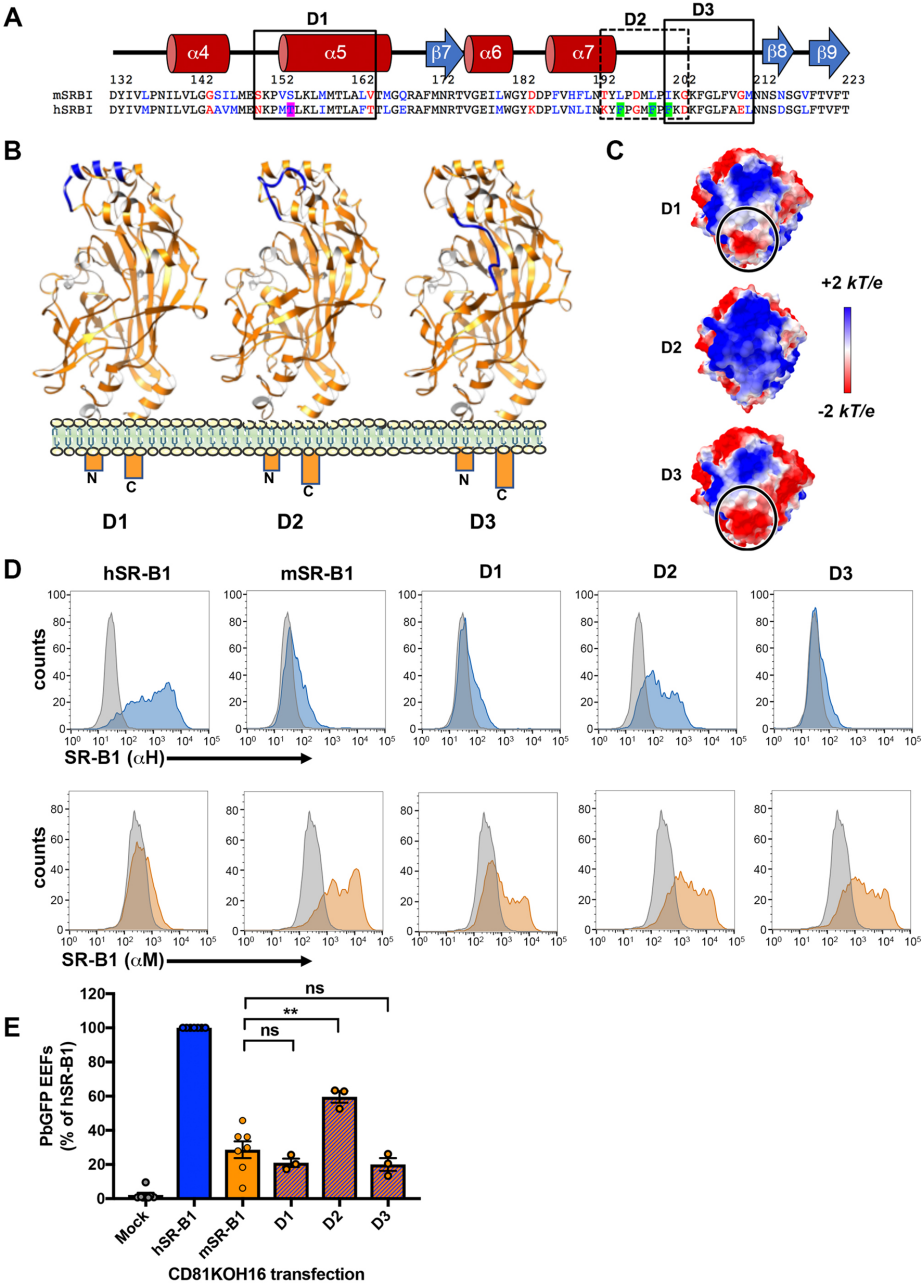
A key domain within the SR-B1 apex regulates *P. berghei* infection. **(A)** Mouse and human protein sequence alignment of the apical region AA 132–223 with the corresponding predicted human secondary structure (alpha helices in red and beta strand in blue). Identical, similar and different amino acids are represented in black, blue and red respectively. Short domains D1, D2 and D3 are delimited by boxes. **(B)** Predicted tertiary structure of D1, D2 and D3 chimeras by homology modeling, highlighting the segments of mouse (orange) or human (blue) origins. **(C)** Top views of the electrostatic surface potential of the D1, D2 and D3 chimera apices. Values are in units of kT/e at 298 K, on a scale of − 2 kT/e (red) to + 2 kT/e (blue). White color indicates a neutral potential. Black circles highlight differential electrostatic surface potentials between the different chimeric constructs at the top of the “apex” region. **(D)** CD81KOH16 cells were transfected with hSR-B1, mSR-B1, D1, D2, or D3 chimeric constructs. Protein surface expression was analyzed using anti-hSR-B1 (“αH”, blue histograms) and anti-mSR-B1 (“αM”, orange histograms), 24 h after transfection. The grey histogram represents untransfected cells stained with the cognate antibody. **(E)** CD81KOH16 cells were transfected with hSR-B1, mSR-B1, D1, D2, or D3 chimeric constructs, or no plasmid as a control (Mock), and then infected with PbGFP sporozoites 24 h after transfection. The number of infected cells (EEFs) was determined by microscopy after UIS4 staining, 24 h after sporozoite addition (mean control values for each experiment: 59, 139, 214, 245, 299, 315 and 383 EEFs/well in hSR-B1-transfected CD81KOH16 cells). ns, non-significant; **p < 0.01 (one-way ANOVA followed by Tukey’s multiple comparisons test).

We then sought to define more precisely the functional regions implicated in *P. berghei* infection within the apex domain. We designed three new chimeras made of a mSR-B1 backbone harboring short hSR-B1 sequences, based on both the amino acid differences between the mouse and the human sequences, and the putative interacting sites in other CD36 family receptors. The D1 chimera (AA 150–164) includes the loop between the α4 and α5 helices, where the Enterovirus 71 interacting site is located in the SR-B1 homolog LIMP-2, and encompasses a large part of the α5 helix including the PfEMP1-interacting site in CD36 (Fig. 6a,b). The D2 chimera (AA 193–203) comprises the external tip of the α7 helix but also three phenylalanine residues in the downstream loop, which are exclusive to the human sequence (Fig. 6a,b). The D3 chimera (AA 201–211) includes only one of these phenylalanine residues (Fig. 6a,b). The predicted electrostatic surface potential of D1 and D3 apex top is similar to mSR-B1 (Fig. 6c), whereas D2 apex is mostly electropositive, like hSR-B1, with no mark of electronegativity.

After the transient transfection of CD81KOH16 cells, D1, D2, and D3 chimeras were all detected by flow cytometry on the cell surface using the anti-mouse “αM” antibody. Interestingly, only D2 was detected by the anti-human “αH” antibody, similarly to hSR-B1 and ApicalH proteins (Fig. 6d). Infection of the transfected cells with *P. berghei* sporozoites revealed that replacement of the AA 193–203 sequence of mSR-B1 by that of hSR-B1 in the D2 chimera resulted in a twofold increase in *P. berghei* infection in CD81KOH16 cells (Fig. 6e). In contrast, replacement of the AA 150–164 or AA 201–211 sequences in the D1 and D3 chimera, respectively, did not increase infection as compared to mSR-B1 (Fig. 6e). These results thus highlight the functional importance of a short 11 amino acid sequence within the hSR-B1 apical domain, which is sufficient to promote efficient *P. berghei* infection.

## Discussion

Previous studies highlighted the dual role of SR-B1 during *Plasmodium* sporozoite invasion and intracellular liver stage development^2, 5^. More recently, we have shown that SR-B1 is an important host factor for *P. vivax* but not for *P. falciparum* infection, and that *P. berghei* sporozoites can use hSR-B1 as an alternative entry route to the CD81-dependent pathway^4^. *P. berghei* is a rodent-infecting parasite, yet *P. berghei* sporozoites can readily infect human cells, using either CD81- or SR-B1-dependent pathways^4^. Here, we show that mouse SR-B1, in contrast to its human counterpart, does not support efficient *P. berghei* sporozoite invasion of murine hepatoma cells.

We took advantage of the differential functionality between human and murine SR-B1 and employed a structure-guided strategy to investigate the SR-B1 molecular determinants involved during *P. berghei* infection. Using complementary chimeras, we demonstrate that the differential ability of human and mouse SR-B1 to support *P. berghei* infection is due to differences in the apex region. The swaps in the chimeras between hSR-B1 and mSR-B1 sequences were engineered at conserved regions and guided by the structure modeling, thus minimizing the risk of incorrect folding of the chimeras. In addition, reciprocal results were observed with the reciprocal chimeras, and both ApicalH and ApicalM constructs mediated HDL binding to cells, suggesting that the chimeric proteins were correctly folded.

The apical helical bundle of the other CD36 family proteins mediates the binding to a variety of ligands. For instance, the ß-glucocerebrosidase binds to LIMP-2 apical domain to be delivered into the lysosome^17^. Binding of Enterovirus 71 depends on a 7 amino acid sequence (AA 144–151) in LIMP-2^18, 19^. Furthermore, an apical phenylalanine of CD36 (F153) binds to *Plasmodium* PfEMP1^7^. These sites can be mapped on the SR-B1 predicted structure at the intersection between the α4 and α5 helices, at the very top of the apex. By analogy with CD36 and LIMP-2, we speculate that the apical helical domain of SR-B1 may serve as a receptor for a hitherto unidentified sporozoite ligand. One candidate is the 6-cysteine domain protein P36, which is required for sporozoite productive invasion of hepatocytes, and is functionally linked to host receptor usage. In particular, we have shown that *P. yoelii* sporozoites genetically complemented with P36 protein from *P. berghei* can infect host cells through a SR-B1-dependent pathway^4^. Whether P36 protein from *P. berghei* or from the medically-relevant *P. vivax* binds to the apical helix bundle of SR-B1 remains to be determined.

In the D2 chimera, a string of 11 residues (AA 193 to 203) in the mouse SR-B1 apical domain, at the end and downstream of the seventh helix, was replaced by the corresponding human sequence. This change significantly increased the ability of mSR-B1 to support *P. berghei* infection. The differential functionality of this small region is consistent with the observation that it is particularly poorly conserved (only 5/11 residues are identical) between the human and mouse sequences. It is possible that this sequence mediates or regulates the interaction with a putative sporozoite ligand. In this regard, and considering the role of CD36 F153 in the binding to *Plasmodium* PfEMP1^7^, it should be noted that the 11 residue string includes 3 phenylalanines present in the human SR-B1 sequence but not in the mouse SR-B1 sequence. In addition, our structure models suggest that the replacement of this sequence by the corresponding human sequence results in a loss of the dense electronegative spot at the apex of mSR-B1. As poorly functional chimeras (ApicalM, D1 and D3) harboured an electronegative apex, and given the apex of hSR-B1 and the functional chimeras (ApicalH and D2) maintained an electropositive site, we speculate that electronegativity conferred by the 11 residue string of mSR-B1 apex is unfavourable for parasite binding. This sequence may reduce the ability of mSR-B1 to support *P. berghei* infection either by directly down-modulating the interaction with a ligand, or indirectly by changing other properties of SR-B1 such as its conformation or the interaction with other host surface molecules.

*P. berghei* sporozoites readily infect CD81-deficient mouse hepatocytes in vivo and in vitro^3, 12^, supporting the existence of alternative entry pathways. Whilst SR-B1 provides a CD81-independent route for *P. berghei* in human cells^4^, we show here that concomitant blockage of murine CD81 and SR-B1 receptors does not prevent *P. berghei* infection in primary mouse hepatocyte cultures. These results support the existence of alternative entry routes for the parasite, which still remain to be identified. Possible candidate host receptors include the SR-B1-related proteins CD36 and LIMP-2. Although LIMP-2 is predominantly expressed in lysosomes, a fraction of the protein pool localizes at the cell plasma membrane, where LIMP-2 acts as a receptor that mediates the Enterovirus 71 host cell entry^19, 20^. LIMP-2 role during *Plasmodium* infection has not been investigated so far. In contrast, CD36 is known to play major roles during malaria infection. CD36 binds PfEMP1 variants expressed at the surface of *P. falciparum*-infected erythrocytes, and contributes to the cytoadherence of *P. falciparum* to vascular endothelial cells^21–23^. It is also a major receptor for tissue sequestration of *P. berghei*-infected erythrocytes in mice^24^. A previous study investigated the contribution of CD36 during *P. yoelii* and *P. berghei* sporozoite infection, using CD36-deficient mice. The data showed that both parasites could still infect hepatocytes in the absence of CD36^25^. However, in these experiments, the presence of a functional CD81-entry pathway could have masked any important role of CD36. Hence the contribution of CD36 and LIMP-2 deserves further investigation.

In conclusion, this study provides new insights into the function of SR-B1 during malaria infection, and paves the way towards a better characterization of the molecular interactions leading to parasite entry into hepatocytes. Our results may be particularly relevant to *P. vivax* malaria, as SR-B1 is the first and up to now only known host entry factor for *P. vivax* sporozoites^4^. The characterization of SR-B1 molecular function and the identification of interacting parasite ligands may lead to the development of novel intervention strategies to prevent *P. vivax* sporozoite entry, before the establishment of the liver stage and the hypnozoite reservoir.

## Methods

### Ethics statement

All animal work was conducted in strict accordance with the Directive 2010/63/EU of the European Parliament and Council on the protection of animals used for scientific purposes. Protocols were approved by the Ethical Committee Charles Darwin N°005 (approval #7475-2016110315516522).

### Experimental animals, parasite and cell lines

We used GFP-expressing *P. berghei* (PbGFP, ANKA strain) parasites, obtained after integration of a GFP expression cassette at the dispensable p230p locus^26^. PbGFP blood stage parasites were propagated in female Swiss mice (6–8 weeks old, from Janvier Labs). *Anopheles stephensi* mosquitoes were fed on PbGFP-infected mice using standard methods^27^, and kept at 21 °C. PbGFP sporozoites were collected from the salivary glands of infected mosquitoes 21–28 days post-feeding. Hepa1-6 cells (ATCC CRL-1830) and HepG2 (ATCC HB-8065) were cultured at 37 °C under 5% CO_2_ in DMEM supplemented with 10% (v/v) fetal calf serum (10500064, Life Technologies), 2 mM l-glutamine (25030024, Life Technologies), and 1% (v/v) of a penicillin–streptomycin solution (15140122, Thermofisher). Primary mouse hepatocytes were isolated by collagenase perfusion (C5138, Sigma), as previously described by Rénia et al.^28^, from C57BL/6 mice harboring a Cre-mediated SR-B1 gene inactivation specifically in the liver^10^. Primary hepatocytes were seeded at confluency in 96 well plates and cultured at 37 °C in 4% CO_2_ in William’s E medium (22551022, Life Technologies) with 10% (v/v) fetal calf serum, 1% (v/v) penicillin–streptomycin (15140122, Life Technologies), 50 µM hydrocortisone (Upjohn laboratories SERB), 2 mM L-glutamine, and 5 µg/ml bovine insulin (I5500, Sigma) for 24 h before sporozoite infection.

### Small interfering RNA silencing of CD81

The siRNA oligonucleotide against CD81 (5′-CGUGUCACCUUCAACUGUA-3′) was validated in previous studies^12^. Transfection of siRNA oligonucleotides was performed by electroporation in the presence of 10 µL of 20 µM siRNA, as previously described^14^. Cells were cultured for 48 h before infection or analysis by immunofluorescence. As negative controls, we used cells electroporated in the absence of siRNA oligonucleotide.

### Generation of a CD81KOH16 cell line using CRISPR-Cas9

The day before transfection, Hepa1-6 cells were plated in 24 well plates at a density of 90 000 cells per well. Cells were transfected with 500 ng of LentiCrispR V2 (Addgene plasmid #52961) containing a guide RNA targeting mouse CD81 (GCAACCACAGAGCTACACCT) using Lipofectamine 2000 (11668027, Life Technologies). Puromycin selection was carried out 36 h after transfection using a 5 µg/ml solution (ant-pr-1, InvivoGen). Cells were exposed to puromycin for 48 h, then washed and expanded for two weeks in DMEM complete medium before analysis. Immunostaining was performed using the rat monoclonal antibody MT81 to label mouse CD81^11^. All incubations were performed at 4 °C for one hour. We used a 2 µg/ml final concentration of Alexa Fluor 488-conjugated Goat anti-rat antibody (A1106, Life technologies) as a secondary antibody. Cells were then fixed with 1% (w/v) formaldehyde solution and analyzed using a Guava EasyCyte 6/2L bench cytometer equipped with 488 nm and 532 nm lasers (Millipore).

### Homology modeling of SR-B1 chimeras

The SR-B1 amino acid sequence of *H. sapiens* (Uniprot: Q8WTV0) was submitted to the HHpred interactive server for remote protein homology detection^29^. The server identified the X-ray structure of the scavenger receptor CD36 (PDB ID: 5lgd) at 2.07 Å resolution^7^ as the best template to model the SR-B1 protein (probability: 100%, e-value: 2.3e−91). Sequences of SR-B1 chimeras were aligned and modeled using Swiss-Model through the ExPAsy molecular biology suite^30^. Each SR-B1 model was then subjected to loop refinement and energy minimization using GalaxyRefine^31^ and YASARA^32^, respectively. SR-B1 models were validated for quality using MolProbity for local stereochemistry^33^, and Prosa II for global 3D quality metrics^34^. Additionally, we validated the structure by checking that all the N-glycosylation sites were solvent-exposed.

The protein electrostatic surface potential was calculated using Adaptive Poisson-Boltzmann Solver (APBS)^35^, after determining the per-atom charge and radius of the structure with PDB2PQR v.2.1.1^36^. The Poisson-Boltzmann equation was solved at 298 K using a grid-based method, with solute and solvent dielectric constants fixed at 2 and 78.5 respectively. We used a scale of −2 kT/e to + 2 kT/e to map the electrostatic surface potential in a radius of 1.4 Å. All molecular drawings were produced using UCSF Chimera^37^.

### SR-B1 chimeric construct design and plasmid transfection

Plasmids encoding human and mouse SR-B1 have been described previously^38, 39^. The ApicalH and ApicalM chimeras were obtained by cloning a single insert amplified from chimeric synthetic genes (Eurofins Genomics) into the mSR-B1 and hSR-B1 plasmids, respectively. The D1, D2 and D3 chimeras were generated by inserting into the mSR-B1 plasmid two fragments amplified with primers containing hSR-B1 sequences. The sequence of all oligonucleotides used to amplify DNA inserts and the sequence of synthetic genes used as templates are indicated in the Supplementary Table S1. The sequence of hSR-B1, mSR-B1 and all chimeric proteins is indicated in the Supplementary Table S2. Information on plasmid sequence is available on demand. All cloning steps were performed using In-fusion cloning kit (639649, Ozyme) and controlled by Sanger sequencing (Eurofins genomics). High concentration plasmid solutions were produced using XL1-Blue Competent Cells (200249, Agilent technology) and plasmid extraction was performed using Qiagen Plasmid Maxikit (12163, QIAGEN) according to the manufacturer’s recommendations. Transfection of SR-B1 or chimeras encoding plasmids was performed 24 h after siRNA electroporation, or directly on CD81KOH16 cells, using the Lipofectamine 2000 reagent (11668027, Life Technologies) according to the manufacturer’s specifications. Following plasmid transfection, cells were cultured for an additional 24 h before sporozoite infection or protein expression analysis.

### Western blot

After cell lysis in 1% (v/v) NP-40, soluble fractions were analyzed by western blot under non-reducing conditions, using a Biorad Mini-Protean electrophoresis chamber for SDS-PAGE and transfer on polyvinylidene fluoride (PVDF) membranes. Membranes were probed with anti-mouse CD81 MT81^11^ at 2 µg/ml, anti-mSR-B1 polyclonal antibody (Ab24603) diluted at 0.9 µg/ml, and anti-mouse GADPH (TAB1001) as a loading control (0.5 µg/ml). Chemiluminescence detection was performed using ECL Prime reagents (RPN2232,GE healthcare Life sciences) and an ImageQuant LAS 4,000 system (GE Healthcare).

### Immunofluorescence assays

For the immunolabeling of SR-B1 and chimeric proteins, cells were harvested using an enzyme-free cell dissociation buffer (13151014, Thermofisher). All incubations were performed at 4 °C in PBS supplemented with 3% (v/v) BSA for one hour with either “αH” anti-SR-B1 polyclonal rabbit serum^39^ or “αM” anti-SR-B1 polyclonal rabbit antibodies NB400-113 (Novus Biological). We used a 2 µg/ml final concentration of Alexa Fluor 488-conjugated Donkey anti-rabbit antibody (Ab150073, Life technologies) as secondary antibody with a 45 min incubation. After fixation in 1% (w/v) formaldehyde, cells were analyzed using a Guava EasyCyte 6/2L bench cytometer equipped with 488 nm and 532 nm lasers (Millipore). Flow cytometry plots are representative of at least three independent experiments.

### In vitro* infection assays*

Hepa1-6 cells were seeded in 96 well plates (2 × 10^4^ per well seeded the day before transfection) and incubated with 1 × 10^4^ PbGFP sporozoites for 3 h, washed, and further cultured until 24 h post-infection. HepG2 and HepG2/CD81, plated in 96 well plates with 3 × 10^4^ cells per well seeded the day before infection, were infected using 5 × 10^3^ PbGFP sporozoites. In some experiments, anti-mouse CD81 MT81 at 20 µg/ml^11^ was added to the cultures at the same time as sporozoites, and the mix was incubated for 3 h before washing the cells with fresh medium. After 24 h, infected cultures were either trypsinized for detection of GFP-positive cells by flow cytometry or fixed with 4% (w/v) paraformaldehyde for fluorescence microscopy. Flow cytometry was performed on a Guava EasyCyte 6/2L bench cytometer (Millipore), and a total of 10,000 cells was analyzed for each sample. For fluorescence microscopy, infected cultures were labeled with polyclonal goat antibodies specific for UIS4 (Sicgen) used at 2 µg/ml, secondary Alexa Fluor 594-conjugated Donkey anti-goat antibodies (A11058, Life technologies) at 2 µg/ml, and the nuclear stain Hoechst 33342. The total number of UIS4-positive EEFs was counted in each well.

### HDL binding assay

Human HDL lipoproteins (LP3, Calbiochem) were labeled using the Cy5 monoreactive Dye pack (PA25001, GE Healthcare) and filtered using Illustra microspin G25 columns (27-532-501, GE Healthcare). CD81KOH16 cells were dissociated at 24 h post-transfection using an enzyme-free cell dissociation buffer (13-151-014, Thermofisher) and incubated with Cy5 labeled HDLs (5 µg/ml) for 20 min at 37 °C. After washing, they were incubated with Suramin (574625, Merck Millipore) at 10 mg/ml for one hour at 4 °C. HDL binding to SR-B1 and chimeras was then evaluated by flow cytometry using the BD LSR Fortessa.

### Statistical analyses

Statistical analyses were performed with GraphPad Prism on at least three independent experiments, each performed at least in triplicate, as indicated in the figure legends. All graphs show the mean ± SEM (unless otherwise indicated) expressed as percentage of control (cells without MT81, WT Hepa1-6 cells or CD81KOH16 cells transfected with hSR-B1, as indicated in the figure legends). Each dot represents the mean of the triplicate values of each experiment.

## Supplementary information

Supplementary Information
