# Supplementary material for: Molecular determinants of SR-B1-dependent Plasmodium sporozoite entry into hepatocytes
Source: Sci Rep. 2020 Aug 11;10:13509. doi: 10.1038/s41598-020-70468-2 (PMC7419504; doi:10.1038/s41598-020-70468-2)
Supplement: Supplementary file 1 — Supplementary Information [file 41598_2020_70468_MOESM1_ESM.pdf]

Supplementary information

Molecular determinants of SR-B1-dependent *Plasmodium* sporozoite entry into hepatocytes.

Anne-Claire Langlois[a], Giulia Manzoni[a], Laetitia Vincensini[a], Romain Coppée[b], Carine Marinach[a], Maryse Guérin[c], Thierry Huby[c], Véronique Carrière[d], François-Loïc Cosset[e], Marlène Dreux[e], Eric Rubinstein[a], Olivier Silvie[a]

[a] Sorbonne Université, INSERM, CNRS, Centre d'Immunologie et des Maladies Infectieuses, CIMI-Paris, F-75013, Paris, France.
[b] Université de Paris, UMR 261 MERIT, IRD, F-75006 Paris, France.
[c] Sorbonne Université, INSERM, Unité de recherche sur les maladies cardiovasculaires, le métabolisme et la nutrition, ICAN, F-75013 Paris, France.
[d] Sorbonne Université, INSERM, Centre de Recherche de St-Antoine, F-75012, Paris, France.
[e] CIRI – Centre International de Recherche en Infectiologie, Univ Lyon, Université Claude Bernard Lyon 1, Inserm, U1111, CNRS, UMR5308, ENS Lyon, F-69007, Lyon, France.

Corresponding author: Olivier Silvie ; olivier.silvie@inserm.fr

Contents

- Supplementary Figures 1 to 3
- Supplementary Table 1
- Supplementary Table 2

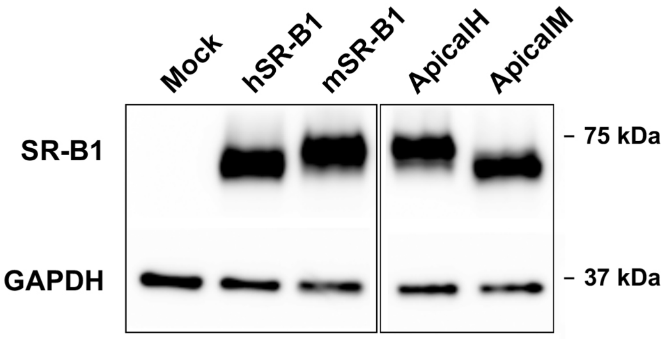

Supplementary Figure 1. CD81 KO Hepa1-6 cells were transfected with either mSR-B1, hSR-B1, ApicalH or ApicalM construct plasmids, or no plasmid as a control (Mock). Total protein expression was analyzed by western blot using polyclonal anti-SR-B1 antibodies (Ab24603) and anti-GAPDH antibodies as a loading control. Full-length blots are shown in Supplementary Figure S3.

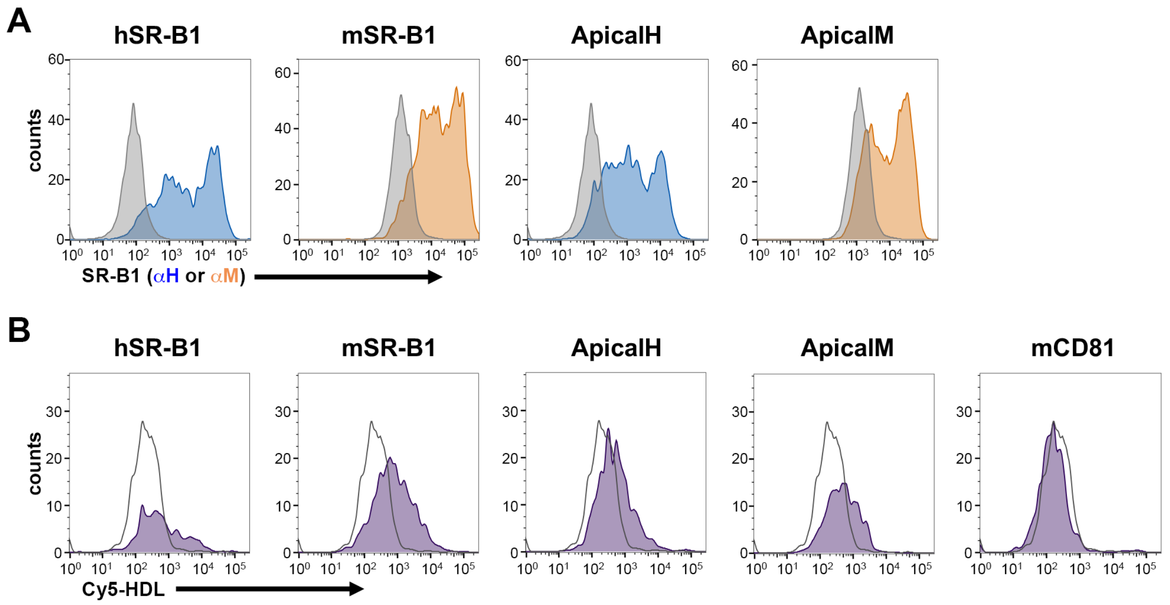

Supplementary Figure 2. **(A-B)** CD81KOH16 cells were transfected with hSR-B1, mSR-B1, ApicalH or ApicalM chimeric constructs, or with a plasmid encoding mCD81 (negative control). **(A)** Protein surface expression was analyzed using anti-hSR-B1 ("αH", blue histograms) and anti-mSR-B1 ("αM", orange histograms), 24 hours after transfection. The grey histogram represents untransfected cells stained with the corresponding antibody. **(B)** Cy5 fluorescent HDLs were added to transfected cells 24 hours after transfection to measure HDL binding (purple peak). HDL binding to non-transfected cells (negative control) is shown as a white peak. Cells transfected with a mouse CD81 construct did not bind HDLs, as expected.

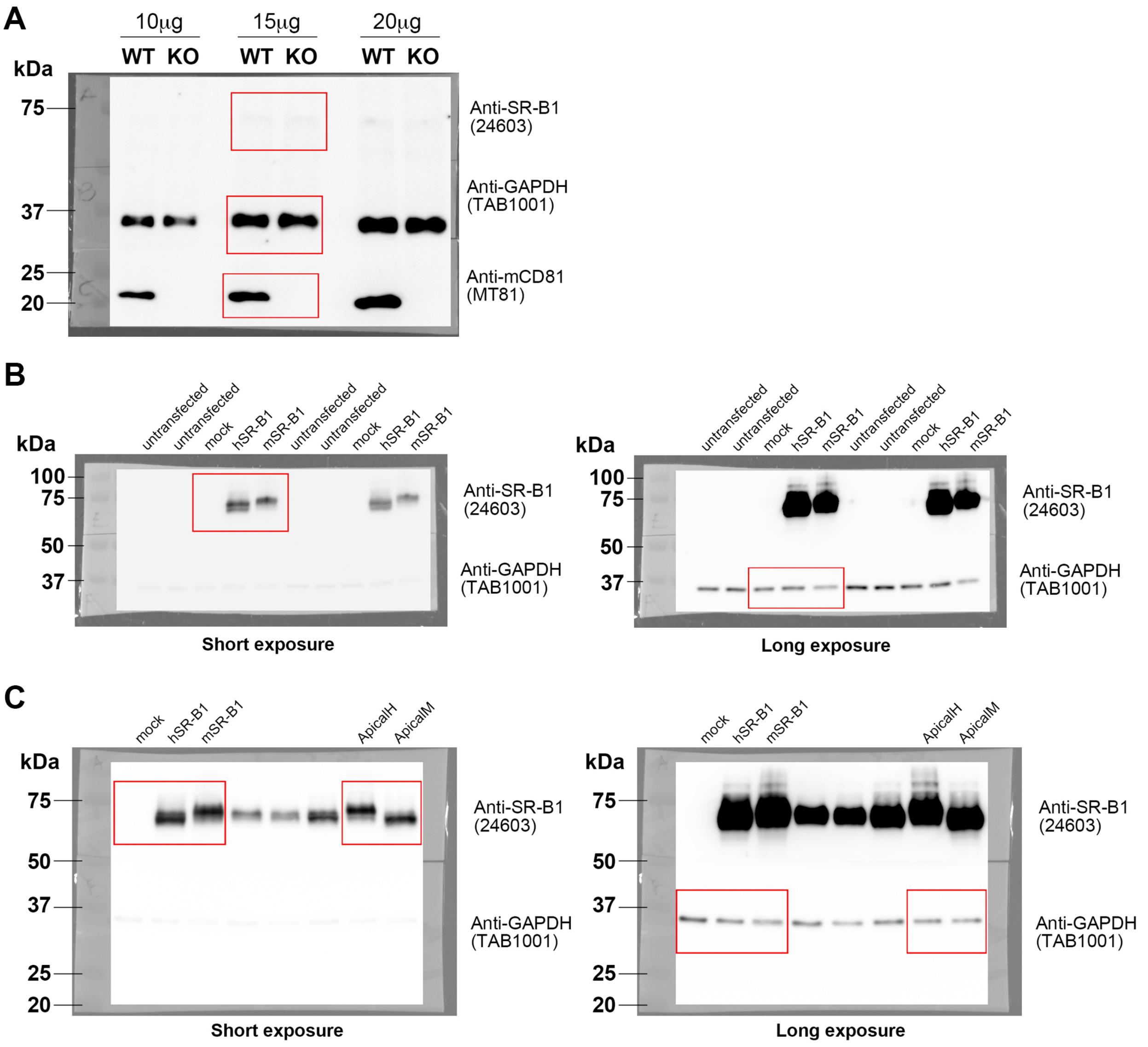

Supplementary Figure 3. **A**. Full-length blot corresponding to Figure 2B. Protein extracts (10µg, 15 µg or 20 µg) were analyzed by SDS-PAGE. After transfer, the membrane was cut into three pieces, which were probed with anti-SR-B1, anti-GADPH and anti-CD81 antibodies, respectively. **B**. Full-length blot corresponding to Figure 3A. Protein extracts from mock, hSR-B1 or mSR-B1 transfected cells were analyzed by SDS-PAGE. After transfer, the membrane was cut into two pieces, which were probed with anti-SR-B1 and anti-GADPH antibodies, respectively. **C**. Full-length blot corresponding to Supplementary Figure S1. Protein extracts from mock, hSR-B1, mSR-B1, ApicalH or ApicalM transfected cells were analyzed by SDS-PAGE. After transfer, the membrane was cut into two pieces, which were probed with anti-SR-B1 and anti-GADPH antibodies, respectively. The red boxes highlight which parts of the full blots are reproduced in the corresponding figures.

Supplementary table 1. Sequences of oligonucleotides and synthetic genes used to assemble the chimeric constructs.

	Oligonucleotide	Sequence 5´ → 3´
**ApicalH construct**	ApicalHfor	ACCGATCCAGCCTCCGCGGGCCCTGC
	ApicalHrev	GAGGCGCACCAAACCTGCAGGTGCTG
**ApicalM construct**	ApicalMfor	CGTGTCCTTCCTCGAGTACCGCACCTTCCAGTTCC
	ApicalMrev	GAGGTGGATCCTCGAGATGTTCTGGACCCCCGTG
**D1 construct**	mSRBIfor	ACCGATCCAGCCTCCGCGGGCCCTGC
	D1rev	CATGATGAGCTTCAGGGTCATGGGCTTATTCTCCATCAATATCGAGCCCCCAG
	D1for	CTGAAGCTCATCATGACCTTGGCATTCACCACGATGGGCCAGCGTGCTTTTATG
	mSRBIrev	GAGGCGCACCAAACCTGCAGGTGCTG
**D2 construct**	mSRBIfor	ACCGATCCAGCCTCCGCGGGCCCTGC
	D2rev	GGGGAACATGCCTGGAAAGTACTTGTTGAGAAAATGCACGAAGGGATCGTC
	D2for	CCAGGCATGTTCCCCTTCAAGGACAAATTTGGCCTGTTTGTTGGGATGAAC
	mSRBIrev	GAGGCGCACCAAACCTGCAGGTGCTG
**D3 construct**	mSRBIfor	ACCGATCCAGCCTCCGCGGGCCCTGC
	D3rev	AAATAATCCGAACTTGTCCTTGAAGGGAAGCATGTCTGGGAGGTACGTG
	D3for	AAGTTCGGATTATTTGCTGAGCTCAACAACTCGAATTCTGGGGTCTTCACTGTC
	mSRBIrev	GAGGCGCACCAAACCTGCAGGTGCTG
**ApicalH synthetic gene**		accgatccagcctccgcgggccctgccaccatgggcggcagctccagggcgcgc<br>tgggtggccttggggttgggcgccctggggctgctgtttgctgcgctcggcgtt<br>gtcatgatcctcatggtgccctccctcatcaagcagcaggtgctcaagaatgtc<br>cgcatagacccgagcagcctgtccttcgggatgtggaaggagatccccgtccct<br>ttctacttgtctgtctacttcttcgaagtggtcaacccaaacgaggtcctcaac<br>ggccagaagccagtagtccgggagcgtggaccctatgtctacagggagttcaga<br>caaaaggtcaacatcaccttcaatgacaacgacaccgtgtccttcgtggagaac<br>cgcagcctccatttccagcctgacaagtcgcatggctcagagagtgactacatt<br>gtactg**CCCAACATCCTGGTCTTGGGTGCGGCGGTGATGATGGAGAATAAGCCC**<br>**ATGACCCTGAAGCTCATCATGACCTTGGCATTCACCACCCTCGGCGAACGTGCC**<br>**TTCATGAACCGCACTGTGGGTGAGATCATGTGGGGCTACAAGGACCCCCTTGTG**<br>**AATCTCATCAACAAGTACTTTCCAGGCATGTTCCCCTTCAAGGACAAGTTCGGA**<br>**TTATTTGCTGAGCTCAACAACTCG**aattctggggtcttcactgtcttcacgggc<br>gtccagaatttcagcaggatccatctggtggacaaatggaacggactcagcaag<br>atcgattattggcattcagagcagtgtaacatgatcaatgggacttccgggcag<br>atgtgggcacccttcatgacacccgaatcctcgctggaattcttcagcccggag<br>gcatgcaggtccatgaagctgacctacaacgaatcaagggtgtttgaaggcatt<br>cccacgtatcgcttcacggcccccgatactctgtttgccaacgggtccgtctac<br>ccacccaacgaaggcttctgcccatgccgagagtctggcattcagaatgtcagc<br>acctgcaggtttggtgcgcctc
**ApicalM synthetic gene**		cgtgtccttcctcgagtaccgcaccttccagttccagccctccaagtcccacgg<br>ctcggagagcgactacatcgtcatg**CCCAACATCCTGGTCCTGGGGGGCTCGAT**<br>**ATTGATGGAGAGCAAGCCTGTGAGCCTGAAGCTGATGATGACCTTGGCGCTGGT**<br>**CACCATGGGCCAGCGTGCTTTTATGAACCGCACAGTTGGTGAGATCCTGTGGGG**<br>**CTATGACGATCCCTTCGTGCATTTTCTCAACACGTACCTCCCAGACATGCTTCC**<br>**CATAAAGGGCAAATTTGGCCTGTTTGCTGAGCTCAACAACTCC**gactctgggct<br>cttcacggtgttcacgggggtccagaacatctcgaggatccacctc

Supplementary Table 2. Protein sequences of hSR-B1, mSR-B1, and chimeras. Sequences of human and murine origin are in blue and orange, respectively. Conserved residues at the chimera boundaries between human and murine SR-B1 are indicated in black.

	Protein sequence
**hSR-B1**	MGCSAKARWAAGALGVAGLLCAVLGAVMIVMVPSLIKQQVLKNVRIDPSSLSFNMWKEIPIPFYLSVYFFDVMN<br>PSEILKGEKPQVRERGPYVYREFRHKSNITFNNNDTVSFLEYRTFQFQPSKSHGSESDYIVMPNILVLGAAVMM<br>ENKPMTLKLIMTLAFTTLGERAFMNRTVGEIMWGYKDPLVNLINKYFPGMFPFKDKFGLFAELNNSDSGLFTVF<br>TGVQNISRIHLVDKWNGLSKVDFWHSDQCNMINGTSGQMWPPFMTPESSLEFYSPEACRSMKLMYKESGVFEGI<br>PTYRFVAPKTLFANGSIYPPNEGFCPCLESGIQNVSTCRFSAPLFLSHPHFLNADPVLAEAVTGLHPNQEAHSL<br>FLDIHPVTGIPMNCSVKLQLSLYMKSVAGIGQTGKIEPVVLPLLWFAESGAMEGETLHTFYTQLVLMPKVMHYA<br>QYVLLALGCVLLLVPVICQIRSQEKCYLFWSSSKKGSKDKEAIQAYSESLMTSAPKGSVLQEAKL
**mSR-B1**	MGGSSRARWVALGLGALGLLFAALGVVMILMVPSLIKQQVLKNVRIDPSSLSFGMWKEIPVPFYLSVYFFEVVN<br>PNEVLNGQKPVVRERGPYVYREFRQKVNITFNDNDTVSFVENRSLHFQPDKSHGSESDYIVLPNILVLGGSILM<br>ESKPVSLKLMMTLALVTMGQRAFMNRTVGEILWGYDDPFVHFLNTYLPDMLPIKGKFGLFVGMNNSNSGVFTVF<br>TGVQNFSRIHLVDKWNGLSKIDYWHSEQCNMINGTSGQMWAPFMTPESSLEFFSPEACRSMKLTYNESRVFEGI<br>PTYRFTAPDTLFANGSVYPPNEGFCPCRESGIQNVSTCRFGAPLFLSHPHFYNADPVLSEAVLGLNPNPKEHSL<br>FLDIHPVTGIPMNCSVKMQLSLYIKSVKGIGQTGKIEPVVLPLLWFEQSGAMGGKPLSTFYTQLVLMPQVLHYA<br>QYVLLGLGGLLLLVPIICQLRSQEKCFLFWSGSKKGSQDKEAIQAYSESLMSPAAKGTVLQEAKL
**ApicalH**	MGGSSRARWVALGLGALGLLFAALGVVMILMVPSLIKQQVLKNVRIDPSSLSFGMWKEIPVPFYLSVYFFEVVN<br>PNEVLNGQKPVVRERGPYVYREFRQKVNITFNDNDTVSFVENRSLHFQPDKSHGSESDYIVLPNILVLGAAVMM<br>ENKPMTLKLIMTLAFTTLGERAFMNRTVGEIMWGYKDPLVNLINKYFPGMFPFKDKFGLFAELNNSNSGVFTVF<br>TGVQNFSRIHLVDKWNGLSKIDYWHSEQCNMINGTSGQMWAPFMTPESSLEFFSPEACRSMKLTYNESRVFEGI<br>PTYRFTAPDTLFANGSVYPPNEGFCPCRESGIQNVSTCRFGAPLFLSHPHFYNADPVLSEAVLGLNPNPKEHSL<br>FLDIHPVTGIPMNCSVKMQLSLYIKSVKGIGQTGKIEPVVLPLLWFEQSGAMGGKPLSTFYTQLVLMPQVLHYA<br>QYVLLGLGGLLLLVPIICQLRSQEKCFLFWSGSKKGSQDKEAIQAYSESLMSPAAKGTVLQEAKL
**ApicalM**	MGCSAKARWAAGALGVAGLLCAVLGAVMIVMVPSLIKQQVLKNVRIDPSSLSFNMWKEIPIPFYLSVYFFDVMN<br>PSEILKGEKPQVRERGPYVYREFRHKSNITFNNNDTVSFLEYRTFQFQPSKSHGSESDYIVMPNILVLGGSILM<br>ESKPVSLKLMMTLALVTMGQRAFMNRTVGEILWGYDDPFVHFLNTYLPDMLPIKGKFGLFAELNNSDSGLFTVF<br>TGVQNISRIHLVDKWNGLSKVDFWHSDQCNMINGTSGQMWPPFMTPESSLEFYSPEACRSMKLMYKESGVFEGI<br>PTYRFVAPKTLFANGSIYPPNEGFCPCLESGIQNVSTCRFSAPLFLSHPHFLNADPVLAEAVTGLHPNQEAHSL<br>FLDIHPVTGIPMNCSVKLQLSLYMKSVAGIGQTGKIEPVVLPLLWFAESGAMEGETLHTFYTQLVLMPKVMHYA<br>QYVLLALGCVLLLVPVICQIRSQEKCYLFWSSSKKGSKDKEAIQAYSESLMTSAPKGSVLQEAKL
**D1**	MGGSSRARWVALGLGALGLLFAALGVVMILMVPSLIKQQVLKNVRIDPSSLSFGMWKEIPVPFYLSVYFFEVVN<br>PNEVLNGQKPVVRERGPYVYREFRQKVNITFNDNDTVSFVENRSLHFQPDKSHGSESDYIVLPNILVLGGSILM<br>ENKPMTLKLIMTLAFTTMGQRAFMNRTVGEILWGYDDPFVHFLNTYLPDMLPIKGKFGLFVGMNNSNSGVFTVF<br>TGVQNFSRIHLVDKWNGLSKIDYWHSEQCNMINGTSGQMWAPFMTPESSLEFFSPEACRSMKLTYNESRVFEGI<br>PTYRFTAPDTLFANGSVYPPNEGFCPCRESGIQNVSTCRFGAPLFLSHPHFYNADPVLSEAVLGLNPNPKEHSL<br>FLDIHPVTGIPMNCSVKMQLSLYIKSVKGIGQTGKIEPVVLPLLWFEQSGAMGGKPLSTFYTQLVLMPQVLHYA<br>QYVLLGLGGLLLLVPIICQLRSQEKCFLFWSGSKKGSQDKEAIQAYSESLMSPAAKGTVLQEAKL
**D2**	MGGSSRARWVALGLGALGLLFAALGVVMILMVPSLIKQQVLKNVRIDPSSLSFGMWKEIPVPFYLSVYFFEVVN<br>PNEVLNGQKPVVRERGPYVYREFRQKVNITFNDNDTVSFVENRSLHFQPDKSHGSESDYIVLPNILVLGGSILM<br>ESKPVSLKLMMTLALVTMGQRAFMNRTVGEILWGYDDPFVHFLNKYFPGMFPFKDKFGLFVGMNNSNSGVFTVF<br>TGVQNFSRIHLVDKWNGLSKIDYWHSEQCNMINGTSGQMWAPFMTPESSLEFFSPEACRSMKLTYNESRVFEGI<br>PTYRFTAPDTLFANGSVYPPNEGFCPCRESGIQNVSTCRFGAPLFLSHPHFYNADPVLSEAVLGLNPNPKEHSL<br>FLDIHPVTGIPMNCSVKMQLSLYIKSVKGIGQTGKIEPVVLPLLWFEQSGAMGGKPLSTFYTQLVLMPQVLHYA<br>QYVLLGLGGLLLLVPIICQLRSQEKCFLFWSGSKKGSQDKEAIQAYSESLMSPAAKGTVLQEAKL
**D3**	MGGSSRARWVALGLGALGLLFAALGVVMILMVPSLIKQQVLKNVRIDPSSLSFGMWKEIPVPFYLSVYFFEVVN<br>PNEVLNGQKPVVRERGPYVYREFRQKVNITFNDNDTVSFVENRSLHFQPDKSHGSESDYIVLPNILVLGGSILM<br>ESKPVSLKLMMTLALVTMGQRAFMNRTVGEILWGYDDPFVHFLNTYLPDMLPFKDKFGLFAELNNSNSGVFTVF<br>TGVQNFSRIHLVDKWNGLSKIDYWHSEQCNMINGTSGQMWAPFMTPESSLEFFSPEACRSMKLTYNESRVFEGI<br>PTYRFTAPDTLFANGSVYPPNEGFCPCRESGIQNVSTCRFGAPLFLSHPHFYNADPVLSEAVLGLNPNPKEHSL<br>FLDIHPVTGIPMNCSVKMQLSLYIKSVKGIGQTGKIEPVVLPLLWFEQSGAMGGKPLSTFYTQLVLMPQVLHYA<br>QYVLLGLGGLLLLVPIICQLRSQEKCFLFWSGSKKGSQDKEAIQAYSESLMSPAAKGTVLQEAKL
